# Selection of reliable reference genes for RT-qPCR analysis during developmental stages and abiotic stress in *Setaria viridis*

**DOI:** 10.1038/srep28348

**Published:** 2016-06-20

**Authors:** Polyana Kelly Martins, Valéria Mafra, Wagner Rodrigo de Souza, Ana Paula Ribeiro, Felipe Vinecky, Marcos Fernando Basso, Bárbara Andrade Dias Brito da Cunha, Adilson Kenji Kobayashi, Hugo Bruno Correa Molinari

**Affiliations:** 1 Genetics and Biotechnology Laboratory, Embrapa Agroenergy (CNPAE), Brasília, DF, 70770-901, Brazil; 2 Brazilian Bioethanol Science and Technology Laboratory/Brazilian Center of Research in Energy and Materials, Campinas, SP, 13083-100, Brazil

## Abstract

Real-time PCR (RT-qPCR) expression analysis is a powerful analytical technique, but reliable results depend on the use of stable reference genes for proper normalization. This study proposed to test the expression stability of 13 candidate reference genes in *Setaria viridis*, a monocot species recently proposed as a new C4 model plant. Gene expression stability of these genes was assayed across different tissues and developmental stages of Setaria and under drought or aluminum stress. In general, our results showed *Protein Kinase*, *RNA Binding Protein* and *SDH* as the most stable genes. Moreover, pairwise analysis showed that two reference genes were sufficient to normalize the gene expression data under each condition. By contrast, *GAPDH* and *ACT* were the least stably expressed genes tested. Validation of suitable reference genes was carried out to profile the expression of *P5CS* and *GolS* during abiotic stress. In addition, normalization of gene expression of *SuSy*, involved in sugar metabolism, was assayed in the developmental dataset. This study provides a list of reliable reference genes for transcript normalization in *S. viridis* in different tissues and stages of development and under abiotic stresses, which will facilitate genetic studies in this monocot model plant.

*Setaria viridis* has emerged as a suitable C4 model species for molecular and genetic studies. It is a short, fast-growing, C4 metabolism plant, with its draft genome sequence recently available, making it suitable model plant for genetic and genomics studies^1^,^2^. Moreover, *S. viridis* is highly responsive to *Agrobacterium tumefaciens*-mediated genetic transformation, with a well-established transformation protocols^1^,^3^ and more recently, spike-dipping methods have also been proposed^3^,^4^. *S. viridis* could be used as a model plant for food and bioenergy grasses presenting C4 metabolism such as maize, sorghum, sugarcane and switchgrass. Genetically engineered *S. viridis* plants can be utilized in a proof-of-concept approach to evaluate phenotypes related to important agricultural traits such as abiotic stress tolerance, resistance to pathogens and improved yield and biomass^5^,^6^,^7^, and the promising genes could be further transferred to a target crop. To achieve this goal, it is important to establish suitable genetic tools, including a reliable gene expression analysis in this species.

Gene expression analysis is an important tool towards the understanding of the complex signaling networks that regulate the different responses observed during the plant life cycle or when they are submitted to different stimulli, and it has been used in many studies for this purpose^8^,^9^,^10^,^11^. Microarray and RNA-sequencing (RNA-seq) are the most widely used technique to provide a global comprehension of gene expression in plants under a wide range of experimental conditions^12^. However, reverse transcription quantitative real-time polymerase chain reaction (RT-qPCR) is the most sensitive, accurate and reproducible technique compared to microarray and RNA-seq to profile the expression levels of genes. Due to these advantages, RT-qPCR has also been used to validate the expression levels of target genes found as differentially expressed by these two methods. However, to avoid biased results during RT-qPCR analysis, a normalization step of the gene expression data is essential to correct variations between different samples and conditions. Normalization during RT-qPCR analysis is usually performed using a reference gene that must be expressed at stable levels regardless of experimental conditions, cell types, tissue, developmental stage or stress treatment^13^. Thus, it is necessary to validate the stability of the reference gene under determined experimental condition to ensure proper normalization and a robust RT-qPCR analysis^14^. Several studies have been published with the aim of identifying suitable reference genes for expression analysis under different stages of the plant life cycle^15^,^16^,^17^,^18^ or under biotic and abiotic stresses^19^,^20^,^21^,^22^.

Abiotic stresses in plants cause major losses in agriculture worldwide. Drought, flooding, extreme temperatures conditions (cold, heat and frost), salinity and mineral toxicity are among the main abiotic stresses that negatively affect growth, development and yield of important crops. The knowledge about the physiology, biochemistry and molecular responses involved in abiotic stresses contributes to the discovery of new genes and signaling networks that plants use to cope with these challenges, and it is pivotal for the development of new crop varieties with enhanced tolerance to stress^23^,^24^,^25^,^26^.

In this study, thirteen genes, *actin* (*ACT*), *anthranilate phosphoribosyl transferase* (*APRT*)*, clathrin adaptor complex* (*CAC*)*, cullin* (*CUL*)*, elongation factor 1-alpha* (*EF1α*)*, eukaryotic initiation factor 4-alpha* (*eIF4α*)*, expressed protein* (*EXP*)*, glyceraldehyde 3-phosphate dehydrogenase* (*GAPDH*)*, protein kinase* (*KIN*)*, RNA-binding protein* (*BIND*)*, RNA polymerase II* (*POL*)*, succinate dehydrogenase* (*SDH*) *and translation initiation factor SUI1* (*SUI*) were selected as candidate reference genes. Besides the traditional genes used for transcript normalization in plants, such as *GAPDH*, *actin* and elongation factors, we selected other genes based on previous reported studies. Different algorithms and statistical analysis were applied to evaluate the expression stability of the reference genes of *S. viridis* plants in different tissues (leaves, stems, spikes and roots) and key developmental stages. In addition, the candidate reference genes were tested to normalize the gene expression in plants submitted to drought stress (*GolS* and *P5CS)* and in different stages of development (*SuSy*), in order to validate the results obtained. It is assumed that the genes analyzed will provide robustness to the gene expression analysis during specific experimental conditions in *S. viridis* plants.

## Results

### Identification of candidate *Setaria viridis* reference genes

**Figure 1:**
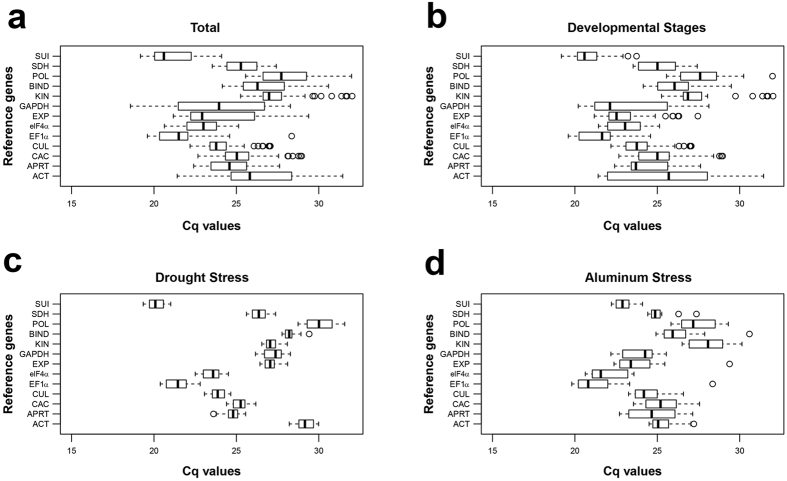
Expression level of reference genes tested in different experimental conditions. Box plot graphs of Cq values are shown as the first and third quartile. Horizontal lines indicate range of values, black lines indicate median values and circles indicate outliers. (**a**) All datasets; (**b**) Developmental stages; (**c**) Drought stress and (**d**) Aluminum stress.

Based on previous systematic studies of reference genes suitable for transcript normalization in monocot^27^,^28^,^29^,^30^,^31^ species, thirteen candidate genes were selected to this study. Information about gene names, accession numbers, primer sequences and efficiency and gene description are provided in Supplementary Table S1. Primers were designed to amplify one single PCR product, as confirmed on a 2% agarose gel and melting curve analysis performed in all RT-qPCR assays (see Supplementary Figs S1 and S2). Mean PCR efficiency per gene was estimated using LinRegPCR (version 2016.0) program; the efficiency values ranged from 89 to 96% for reference genes and 90 to 100% for target genes (see Supplementary Table S1). Expression levels of the candidate genes for different developmental stages, drought and aluminum stresses and for all samples combined are presented in Fig. 1. Expression values are inversely proportional to the Cq values, and the mean and range of Cq indicate the most stable genes across all samples and to each experimental set. Cq values of candidate genes from each experimental set presented a high variation, ranging from 18.6 for *GAPDH* and 32.0 for *POL*. Expression levels to each subset of the candidate genes for different tissues/organs can be found in Supplementary Fig. S3.

### Expression stability analysis

Performance of the thirteen genes as potential reference genes for *S. viridis* was assessed in 31 samples divided into three experimental sets; five developmental stages from whole seedlings, different tissues/organs, and two treatments, including samples submitted to different levels of drought or aluminum stresses. Using geNorm, we estimated two parameters to evaluate the expression stability of these genes; the average expression stability value (*M* value), based on the pairwise variation between a particular gene compared to all others, and the pairwise variation (Vn/n + 1), which determines the required number of genes to result in a more accurate normalization^32^.

**Figure 2:**
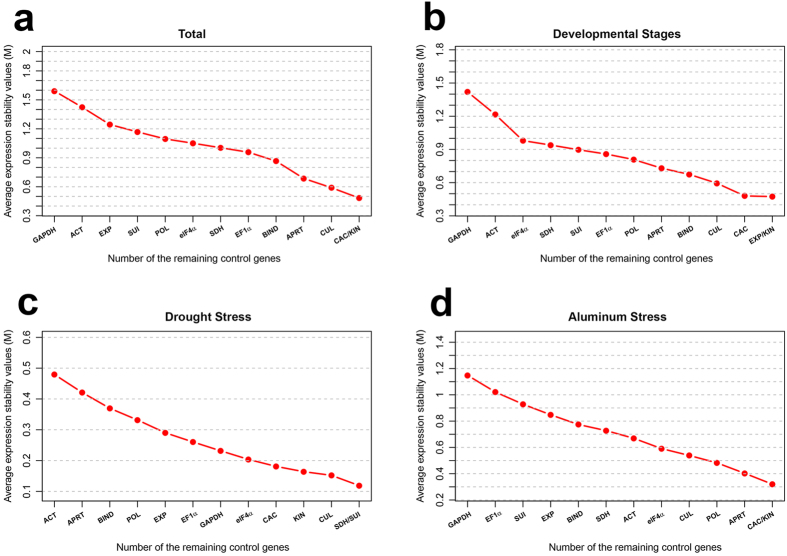
Average expression stability values (M) calculated by geNorm. A lower value of average expression stability (M) indicates most stable expression. (**a**) All datasets; (**b**) Developmental stages; (**c**) *Setaria viridis* submitted to drought and (**d**) aluminum stress.

When considering all dataset, *CAC*/*KIN* (M = 0.48) was the best pair to normalize all samples, while *GAPDH* was the least stable gene (M = 1.6) (Fig. 2a). Comparing with NormFinder, *SDH* was the most stable gene and *CUL*/*KIN* were defined as the best pair for a reliable normalization. In both programs, *ACT* and *GAPDH* were ranked as the least stable genes (Tables 1 and 2).

Due to the heterogeneity of these samples and conditions, each experimental set was analyzed individually using both algorithms. While geNorm performs a stepwise exclusion of the least stably expressed gene^32^, NormFinder uses a model-based approach, which calculates both inter- and intra-group variability to estimate the stability of gene expression^33^. Estimative of the best reference genes in each experimental set exhibited some particularities. For developmental stages, *EXP/KIN* pair (M = 0.47) was ranked as the most stable gene pair, while *BIND* was the most stable gene by NormFinder, followed by *SDH* (Tables 1 and 2; Fig. 2b). Once again, *ACT*/*GAPDH* showed the highest variation and hence, they were not suitable for normalization in different stages of development (Tables 1 and 2). We also analyzed the expression stability of these candidate genes in samples derived from whole seedlings in vegetative phase and in each tissue/organ at the subsequent stages of development. geNorm and NormFinder excluded the same reference genes, but defined different pair of genes as the best reference genes to each particular subset of tissue/organ. In general, *SDH* and *eIF4α* were selected as the preferred reference genes when considering both algorithms (see Supplementary Tables S2 and S3; Supplementary Fig. S4).

For drought treatment, *SDH/SUI* pair and *SDH* gene were considered the most stable genes according to geNorm and NormFinder, respectively. The best pair according to NormFinder was *KIN*/*CUL*, whereas *ACT* was estimated as the most variable reference gene by both algorithms (Fig. 2c; Tables 1 and 2).

For aluminum treatment, *CAC/KIN* pair presented the best performance, according to geNorm (Fig. 2d and Table 1). Although *CUL* was the most stable, according to NormFinder, *CAC* was ranked in the top-three position (Table 2). In both geNorm and NormFinder, *EXP*, *SUI*, *EF1α* and *GAPDH* showed the highest variation among all the reference genes tested under Al^3+^ treatment (Tables 1 and 2).

**Figure 3:**
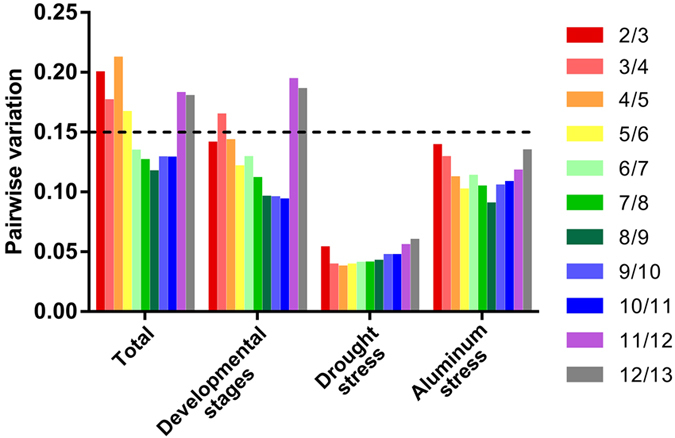
Pairwise variation (V) to define the optimal number of reference genes required to a reliable normalization to each dataset.

In addition, to define the best pair using geNorm, we also estimated the pairwise variation to determine the minimal number of genes for reliable normalization. Assuming a cut-off of Vn/n + 1 ≤ 0.15, it was determined that the use of only the top two reference genes for each experimental set would be the appropriate number of genes required for normalization (Fig. 3; Supplementary Fig. S5). When the entire dataset were considered, the number of genes increased to six (Fig. 3).

### Validation of the selected reference genes to different experimental conditions

**Figure 4:**
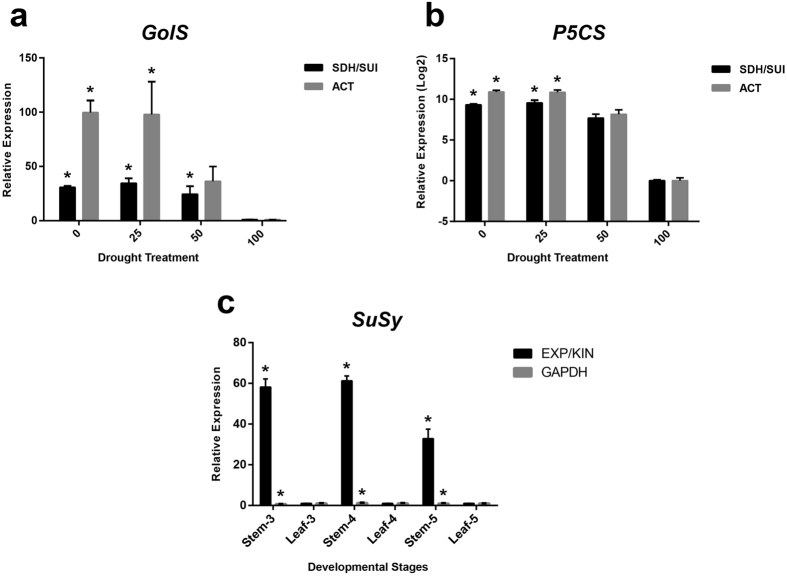
Relative expression level of target genes using the most and least stable pair of reference genes to each experimental condition, as determined by geNorm. (**a**,**b**) Transcription levels of *GolS* and *P5CS* genes in *Setaria viridis* submitted to drought stress treatment (0 (Ψ_w_-1.50 MPa; permanent wilting point), 25 (Ψ_w_-1.125 MPa; severe stress), 50 (Ψ_w_-0.75 MPa; moderate stress) and 100% (Ψ_w_-0.03 MPa; field capacity), respectively; (**c**) Transcription levels of *SuSy* in tree developmental stages. Bars indicate the standard error (±SE) calculated from three biological replicates. The asterisks indicate statistically significant with respect to control (ANOVA followed by Tukey’s test).

In order to validate the selection of reference genes in *S. viridis* under drought treatment, the expression levels of *GolS* and *P5CS*, two marker genes for drought stress, were normalized to the best pair (*SDH/SUI*) or the most variable reference gene (*ACT*), according to geNorm (Fig. 2c). Normalization of transcripts using *SDH/SUI* showed that transcript levels increased significantly upon 0 (Ψ_w_-1.50 MPa; permanent wilting point), 25 (Ψ_w_-1.125 MPa; severe stress) and 50% (Ψ_w_-0.75 MPa; moderate stress) of soil water content (SWC) for *GolS* (Fig. 4a) and under 0 and 25% of SWC for *P5CS* (Fig. 4b), when compared to 100% of SWC (Ψ_w_-0.03 MPa; field capacity). These results were expected, since *P5CS* and *GolS* genes are known to increase their expression levels under drought conditions in plants^34^,^35^,^36^. By contrast, normalization using *ACT* as reference gene resulted in an overestimated relative expression level of both target genes (Fig. 4a,b). In addition, relative expression of *GolS* (normalized to *ACT*) during moderate stress was not significantly different compared to control (Fig. 4a).

Similarly, top ranked reference genes samples for developmental stages datasets were validated using *SuSy* gene, responsible for sucrose synthase production and involved in the sucrose metabolism in plants. *SuSy* expression is known to increase during the plant life cycle in C4 species such as sugarcane and sorghum^37^,^38^,^39^. The increase of *SuSy* expression levels during the late stages of *S. viridis* development was confirmed using *EXP*/*KIN*, the best pair of reference genes for the developmental stages dataset (Fig. 4c). When *GAPDH*, the least stable gene for developmental stages dataset, was used to normalize *SuSy* expression, no significant difference was observed.

## Discussion

RT-qPCR has emerged as the standard method for gene expression profiling due to its high sensibility, reproducibility and large dynamic range with a potential increasing sample throughput^13^,^40^. However, a reliable RT-qPCR quantification assay depends upon the selection of stable reference genes for a proper normalization of the expression levels measured^41^. Suitable reference genes should be stably expressed in all samples and experimental conditions under evaluation. Therefore, many studies have been conducted to provide a systematic selection of reliable reference genes candidates to different plant species^15^,^17^. In *S. viridis*, a recent study reported the selection and validation of fifteen reference genes. They found gene that encoded a phosphoglucomutase, folylpolyglutamate synthase and cullin as the most stable reference genes. However, this study was focused on the validation of reference genes only for a leaf gradient dataset and stages of development^17^. In the present study, we selected thirteen candidate genes in *S. viridis* to be tested in a total of 112 samples, comprising three experimental sets: developmental stages, different tissues/organs and two abiotic (drought and aluminum) stresses. The selected candidate genes were chosen based on previous studies that identified suitable reference genes in monocot species^27^,^28^,^29^,^30^,^31^.

We were able to carry out two simple methods of RNA extraction that yielded a high quality and quantity for all samples. RNA from most tissues/organs of *S. viridis* was extracted using the TRIzol method (Thermo Scientific), except for RNA from roots, which was extracted using a LiCl method^42^. The SYBR Green detection dye^13^ was used in the RT-qPCR for transcript detection and the results obtained were analyzed by different algorithms to verify the stability of the candidate genes expression.

Analysis in both geNorm and NormFinder showed some differences in the top-ranked genes but both programs were more consistent to exclude the least stable genes. These discrepancies reflect differences between the approaches^33^. The difference was more evident in the developmental stages dataset, possibly due to the higher heterogeneity of samples assessed in each subset (different tissues/organs and developmental stages), when compared to the abiotic stress conditions (treated *versus* untreated samples). Since NormFinder estimates both inter- and intra-group variation and combines them into a stability value, this model-based approach should provide a more precise and robust estimative of expression variation among subsets composed by different sample types^33^. Differences in the top-ranked genes selected by both methods were also reported in different tissues, flower stages and fruit development in cotton^43^, fruit development in apple^44^ and different organs and flower developmental stages in citrus^45^. In this study, the gene pairs *EXP*/*KIN* and *BIND*/*SDH* were considered the most suitable pair of genes to normalize samples in different developmental stages, according to geNorm and NormFinder, respectively. Concerning different tissues/organs and both algorithms, *SDH*/*elF4α* was ranked as the best gene pair for a proper normalization.

For drought stress, geNorm and NormFinder identified the same four top-ranked genes with few differences in some positions. *SDH*/*SUI* was the best pair of reference genes according to geNorm. *SDH* was the most stable gene, although *CUL/KIN* was the best pair, according to NormFinder. *SDH* gene was stable for normalization of genes in *Brachypodium distachyon* under different developmental stages^30^. *SUI* and *KIN* showed stable expression in different tissues, development and under biotic and abiotic stresses in rice^31^. The member of the cullin family *CUL* has been reported as the most stable gene in leaf gradient dataset to *S. viridis*^17^ and one of the three-top genes stably expressed in sugarcane under salinity or drought stress^27^. *APRT* and *ACT* were the most variable reference genes under drought stress in our study, thereby they were considered inappropriate to use as a reference gene under this condition. Accordingly, *APRT* was also found unstable in sugarcane under abiotic stress conditions^28^, whereas *ACT* was selected as a suitable reference gene to switchgrass under drought and salinity treatment^46^, demonstrating that even phylogenetically similar species show differential responses to gene expression under the same experimental conditions.

Considering Setaria seedlings submitted to aluminum stress, we found by geNorm that *CAC* and *KIN* were the most stable reference genes tested, although *CAC* was only ranked as the third position, according to NormFinder. In contrast, four genes (*EXP*, *SUI*, *EF1a* and *GAPDH*) changed significantly in their expression levels and then should be carefully evaluated before using them to normalize the expression of target genes in *S. viridis* under similar stress conditions. Unlike to drought or other abiotic stresses in which several studies reported the selection of reference genes in different plant species^29^,^47^,^48^,^49^ to the best of our knowledge, this is the first report describing the validation of reference genes for aluminum stress condition in plants.

**Figure 5:**
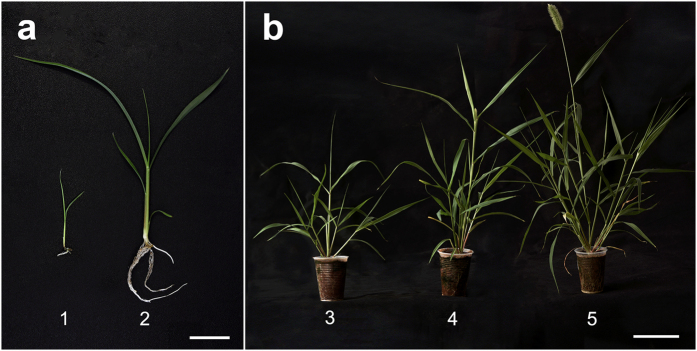
Developmental stages of *Setaria viridis* used in this study. (**a**) 1 - early vegetative phase (EVP) and 2 - late vegetative phase (LVP), bar = 2 cm; (**b**) 3 - transition phase (TP), 4 - reproductive phase (RP) and 5 - advanced phase (AP), bar = 10 cm.

The suitability of the top ranked genes for the normalization of transcript studies under different stages of development of Setaria was performed by studying the expression of a sucrose synthase gene (*SuSy*). Our previous results (unpublished data) demonstrated that *SuSy* (Sevir.4G039300) expression increased in the late stages of *S. viridis* development, and such expression is accompanied by high levels of sucrose in the culms of the plant in the same developmental stage (data not shown). According to geNorm, the most suitable gene pair for the transcript normalization in different developmental stages was *EXP*/*KIN* (Table 1). In fact, using *EXP*/*KIN* to normalize *SuSy* expression in three different stages of development, we were able to demonstrate that the expression of this gene increased as the plant reached the last stage (Figs 4c and 5). In contrast, using the least stable gene *GAPDH* was not suitable to normalize *SuSy* expression in the different plant developmental stages. We also performed the normalization of the expression of two drought marker genes, *PC5S* and *GolS*, using *SDH*/*SUI* as reference genes, suggested as the most stable pair of genes under drought stress according to geNorm. The *P5CS* gene codifies for Δ^1^-pyrroline-5-carboxylate synthetase, which catalyzes the rate-limiting step in the biosynthesis of proline, an amino acid that accumulates in plants as the water deficit becomes more severe^50^,^51^. *GolS* is the gene responsible for the transcription of galactinol synthase, a key enzyme involved in raffinose family oligosaccharide biosynthesis, which is highly expressed under abiotic stress conditions^35^,^52^,^53^. Our results demonstrated the reliability of *SDH*/*SUI* as reference genes to normalize the transcription of *P5CS* and *GolS*, as the expression levels of the marker genes increased as the water deficit becomes more severe (Fig. 4a,b). These results were not observed when *ACT*, the least stable reference gene for drought stress, was used for normalization.

In summary, we found that the stability of expression of reference genes varied depending on the experimental dataset tested. In general, *KIN*, *BIND* and *SDH* were the best ranked reference genes considering developmental set and drought stress treatments. In contrast, traditional genes such as *GAPDH* and *ACT* varied significantly among our conditions. Different tissues, developmental stages and even different abiotic stresses could influence the expression stability of reference genes. This was more evident when we evaluated the expression stability of reference genes to each tissue or developmental stage individually. In addition, validation of target genes *P5CS* and *GolS* normalized to the best pair of reference genes or the most variable gene showed that the arbitrary use of a reference gene without a prior selection could lead to a misinterpretation of data. These results reinforce the need of a systematic evaluation of appropriate reference genes to each particular condition to improve the reliability of gene expression assays and avoid biased results. In this sense, this study provides a list of suitable *S. viridis* reference genes for validation procedures.

## Material and Methods

### Plant material and growth conditions

Seeds of *S. viridis* (accession A10.1) were treated with concentrated sulfuric acid to promote dormancy break^3^. After the mature seeds were germinated on half strength MS medium^54^ in growth chamber (Conviron) under 16 h photoperiod, 25 ± 2 °C and light intensity of 150 μmol m^−2^s^−1^ for ten days (considered early vegetative phase). After this period, *S. viridis* seedlings were transferred to pots containing latosoil, substrate (Plantmax) and vermiculite (Agrifloc, Brasil Minérios) mixture (3:1:0.5; w/w/w). Plants were maintained in growth chamber (Fitotron) under 16 h photoperiod of 500 μmol m^−2^s^−1^ light intensity, 26 ± 2 °C and 65% relative humidity.

### *S. viridis* tissues, organs and developmental stages

*S. viridis* seedlings were harvested at the early vegetative phase (EVP) 10 days after *in vitro* germination and the late vegetative phase (LVP) 7 days after planting (DAP). In the transition (TP; 25 DAP) and reproductive (RP; 32 DAP) phases, the plants were separated into leaf, stem and root tissues. Spikes and other organs were harvested in the advanced phase (AP; 39 DAP) (Fig. 5). Pools of three whole seedlings or tissues were harvested comprising three biological samples. Samples were transferred to liquid nitrogen and stored at −80^◦^ C until analyses.

### Drought treatment

Plants at the reproductive phase (32 DAP) were maintained in growth chamber in the same conditions described above. Four levels of soil water content (SWC) were used, 0 (Ψ_w_-1.50 MPa; permanent wilting point), 25 (Ψ_w_-1.125 MPa; severe stress), 50 (Ψ_w_-0.75 MPa; moderate stress) and 100% (Ψ_w_-0.03 MPa; field capacity). The net photosynthetic rate was assessed to characterize the water stress using an open gas exchange system with a 6 cm^2^ clamp-on leaf cuvette (LI-6400XT, LICOR). Photosynthetic photon flux density (PPFD) was fixed at 1,500 μmol m^−2^ s^−1^, using a red-blue LED light source built into the leaf cuvette. Twenty-four hours after the permanent wilting point, whole +2 leaves (second fully expanded leaf with visible ligule) were harvested from six plants and pooled composing each of the three biological samples. Collected tissues were frozen immediately in liquid nitrogen and stored at −80 °C.

### Aluminum treatment

Seedlings in early vegetative phase were submitted to 500 μM CaCl_2_ solution, in the absence or presence of {20} μM Al^3+^, pH 4.2, in hydroponic system for 24 hours. Roots of 120 plantlets were harvested, separated in three biological replicates (40 each) and immediately frozen in liquid nitrogen and stored at −80 °C.

### Selection of candidate reference genes and primer design

Thirteen candidate reference genes were selected in the present study using as criterion reference genes previously reported as suitable for transcript normalization in other monocots^27^,^28^,^29^,^30^,^31^ subject to different experimental conditions. Initially, we used the *Setaria italica* genome database (Phytozome 10.1 v) as reference to retrieve the ortholog gene sequences. With the recent release of the *S. viridis* genome sequence at Phytozome database, we confirmed the identity and specificity of primer sequences used in this study. Using BLASTN algorithm with a default setting and *S. italica*, *Oryza sativa*, sugarcane and *B. distachyon* sequences as queries, *Setaria* spp. coding sequences with high similarity scores were retrieved (E-value ≤ 1e-90) (see Supplementary Table S1). Primers were designed using Primer Express 3.0 (Applied Biosystems) and PrimerQuest (IDT) tools with the following parameters: T_m_ around 60 °C and amplicon length of 75 to 150 bp, yielding primer sequences with a length of 19 to 23 nucleotides with an optimum at 20 nucleotides, and a GC content of 45 to 60%. Primers were also designed to span exon-exon junction and allow the amplification of all splicing variants.

### RNA isolation and cDNA synthesis

About 200 mg of starting material was used for RNA isolation. Total RNA from all tissues (except roots) was isolated using TRIzol Reagent (Thermo Scientific), according to the manufacturer’s instructions. RNA from roots was extracted using a LiCl method^42^. Genomic DNA was removed using RQ1 RNase-free DNase (Promega), according to the manufacturer’s instructions. Total RNA was quantified using a NanoDrop ND-1000 Spectrophotometer (Uniscience), and RNA integrity was verified in agarose gel electrophoresis. Reverse transcription reaction was carried out with 1 μg of total RNA and oligo (dT) in a total volume of 20 μL using RevertAid First Strand cDNA Synthesis Kit (Thermo Scientific), following the manufacturer’s recommendations. cDNA samples were diluted (1:25) prior to use in RT-qPCR assays.

### Quantitative Real-time PCR conditions

RT-qPCR was carried out in a 96-well optical plate with a StepOnePlus Real-Time PCR Systems (Applied Biosystems). Reactions were performed using Platinum SYBR Green PCR SuperMix-UDG with ROX (Invitrogen), 0.2 μM of each primer and 1 μL of diluted cDNA (1:25) in a final volume of 10 μL. The following thermal cycling condition was used for all amplifications: 2 min at 50 °C min, 20 sec at 95 °C, followed by 40 amplification cycles of 95 °C for 3 sec, and 60 °C for 30 sec. After 40 cycles, the specificity of the amplifications was analyzed through the dissociation curve profiles. Quantification cycle threshold (Cq) values per target were manually estimated. Background-corrected raw fluorescence data were imported into LinRegPCR version 2016.0 software for primer efficiency estimation^55^. The program uses linear regression analysis to fit a straight line and estimate PCR efficiency of each individual sample based on the slope of this line^56^,^57^. All assays were performed using three biological replicates with three technical replicates each and a non-template control.

### Assessing the expression stability of reference genes

To estimate the expression stability of reference genes, Cq values were converted into non-normalized relative quantities. These values are obtained using the formula Q = E^ΔCq^, where E represents the average efficiency for each gene, and ΔCq represents the difference between the lowest Cq value of a sample of a particular gene and the Cq value of each sample in a dataset^40^. These data were imported into R/Bioconductor for reference genes selection using geNorm (medgen.ugent.be/∼jvdesomp/geNorm/)^32^ and NormFinder ( www.mdl.dk/publicationsNormFinder.htm)^33^ algorithms. Global analysis was performed using all datasets. Subsequently, each experimental set was assessed to define specific reference genes for proper normalization.

### Validation of reference genes

Validation of the selected reference genes was carried out in samples of drought treatment and developmental series. In drought treatment, we analyzed the expression pattern of *P5CS* and *GolS*, two genes that function as osmoprotectants in drought-stress tolerance in plants^34^,^35^. We also quantified the relative expression of *SuSy* in stem compared to leaf tissues during *S. viridis* development. Normalization of both target genes was performed using either the two most stable candidate reference genes or the least stable one as determined by geNorm analysis.

## Additional Information

**How to cite this article**: Martins, P. K. *et al*. Selection of reliable reference genes for RT-qPCR analysis during developmental stages and abiotic stress in *Setaria viridis*. *Sci. Rep.* **6**, 28348; doi: 10.1038/srep28348 (2016).

## Supplementary Material

Supplementary Information

## Figures and Tables

**Table 1:** *Setaria viridis* reference genes ranked according to expression stability as determined by geNorm.

**Ranking**	**Total**		**Developmental Stages**		**Drought Stress**		**Aluminum Stress**	
	**geNorm**	**Stability value**	**geNorm**	**Stability value**	**geNorm**	**Stability value**	**geNorm**	**Stability value**
1	*CAC*	0.48	*EXP*	0.47	*SDH*	0.12	*CAC*	0.32
2	*KIN*	0.48	*KIN*	0.47	*SUI*	0.12	*KIN*	0.32
3	*CUL*	0.59	*CAC*	0.48	*CUL*	0.15	*APRT*	0.40
4	*APRT*	0.69	*CUL*	0.59	*KIN*	0.16	*POL*	0.48
5	*BIND*	0.87	*BIND*	0.67	*CAC*	0.18	*CUL*	0.54
6	*EF1α*	0.96	*APRT*	0.73	*eIF4α*	0.20	*eIF4α*	0.59
7	*SDH*	1.00	*POL*	0.81	*GAPDH*	0.23	*ACT*	0.67
8	*eIF4α*	1.05	*EF1α*	0.86	*EF1α*	0.26	*SDH*	0.73
9	*POL*	1.10	*SUI*	0.90	*EXP*	0.29	*BIND*	0.77
10	*SUI*	1.17	*SDH*	0.94	*POL*	0.33	*EXP*	0.85
11	*EXP*	1.24	*eIF4α*	0.98	*BIND*	0.37	*SUI*	0.93
12	*ACT*	1.42	*ACT*	1.22	*APRT*	0.42	*EF1α*	1.02
13	*GAPDH*	1.59	*GAPDH*	1.42	*ACT*	0.48	*GAPDH*	1.15
Best pair	*CAC/KIN*		*EXP/KIN*		*SDH/SUI*		*CAC/KIN*	

**Table 2:** *Setaria viridis* reference genes ranked according to expression stability as determined by NormFinder.

**Ranking**	**Total**		**Developmental Stages**		**Drought Stress**		**Aluminum Stress**	
	**NormFinder**	**Stability value**	**NormFinder**	**Stability value**	**NormFinder**	**Stability value**	**NormFinder**	**Stability value**
1	*SDH*	0.06	*BIND*	0.06	*SDH*	−0.00097	*CUL*	0.02
2	*BIND*	0.10	*SDH*	0.09	*KIN*	−0.00066	*ACT*	0.07
3	*CAC*	0.29	*EF1α*	0.10	*CUL*	0.00001	*CAC*	0.10
4	*eIF4α*	0.34	*eIF4α*	0.22	*SUI*	0.01	*SDH*	0.10
5	*EF1α*	0.37	*CAC*	0.25	*CAC*	0.01	*POL*	0.13
6	*POL*	0.38	*EXP*	0.29	*EXP*	0.03	*BIND*	0.15
7	*CUL*	0.44	*SUI*	0.29	*eIF4α*	0.04	*KIN*	0.23
8	*APRT*	0.45	*POL*	0.33	*GAPDH*	0.07	*eIF4α*	0.32
9	*KIN*	0.57	*CUL*	0.44	*BIND*	0.07	*APRT*	0.35
10	*EXP*	0.78	*APRT*	0.46	*EF1α*	0.09	*EXP*	0.47
11	*SUI*	0.94	*KIN*	0.49	*POL*	0.15	*SUI*	0.57
12	*ACT*	1.94	*ACT*	2.25	*APRT*	0.17	*EF1α*	0.99
13	*GAPDH*	2.56	*GAPDH*	2.75	*ACT*	0.28	*GAPDH*	1.43
Best pair	*SDH/BIND*		*BIND/SDH*		*KIN/CUL*		*CUL/BIND*	
