# Supplementary material for: Selection of reliable reference genes for RT-qPCR analysis during developmental stages and abiotic stress in Setaria viridis
Source: Sci Rep. 2016 Jun 20;6:28348. doi: 10.1038/srep28348 (PMC4913262; doi:10.1038/srep28348)
Supplement: Supplementary Information [file srep28348-s1.pdf]

Selection of reliable reference genes for RT-qPCR analysis during developmental stages and abiotic stress in *Setaria viridis*

Polyana Kelly Martins[1], Valéria Mafra[2], Wagner Rodrigo de Souza[1], Ana Paula Ribeiro[1], Felipe Vinecky[1], Marcos Fernando Basso[1], Bárbara Andrade Dias Brito da Cunha[1], Adilson Kenji Kobayashi[1] and Hugo Bruno Correa Molinari[1*]

[1]Genetics and Biotechnology Laboratory, Embrapa Agroenergy (CNPAE), Brasília, DF, 70770-901, Brazil.

[2]Brazilian Bioethanol Science and Technology Laboratory/Brazilian Center of Research in Energy and Materials, Campinas, SP, 13083-100, Brazil.

*Corresponding author: Phone: +55 61 3448 2307; +55 61 3448 1589. E-mail address: hugo.molinari@embrapa.br

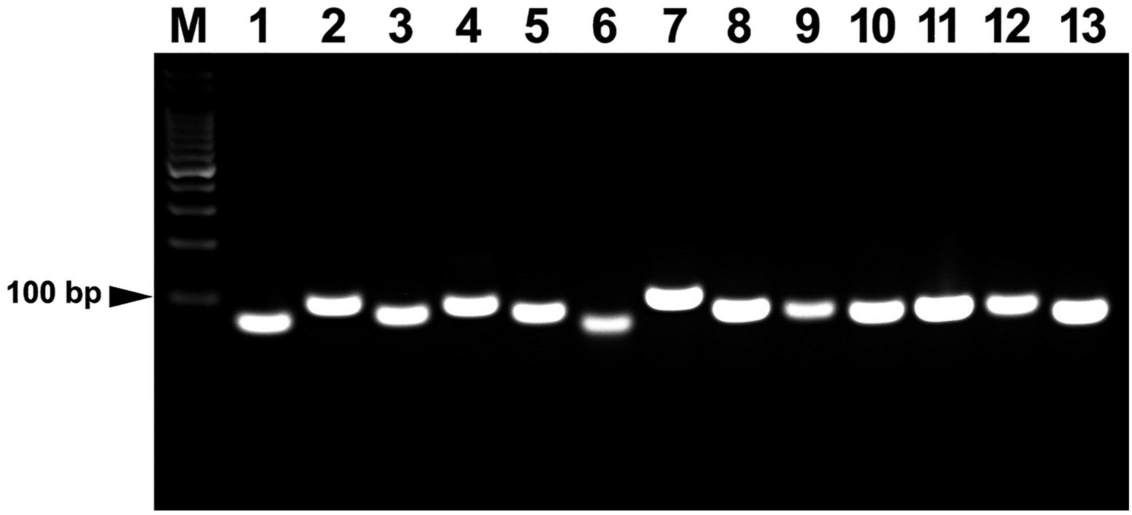

Figure S1: RT-qPCR amplification specificity of the 13 reference genes. Amplification fragments were separated by 2% agarose gel electrophoresis. 1 – *elF4α*, 2 – *ACT*, 3 – *SDH*, 4 – *CAC*, 5 – *CUL*, 6 – *POL*, 7 – *APRT*, 8 – *EXP*, 9 – *BIND*, 10 – *KIN*, 11 – *GAPDH*, 12 – *SUI* and 13 – *EF1α*.

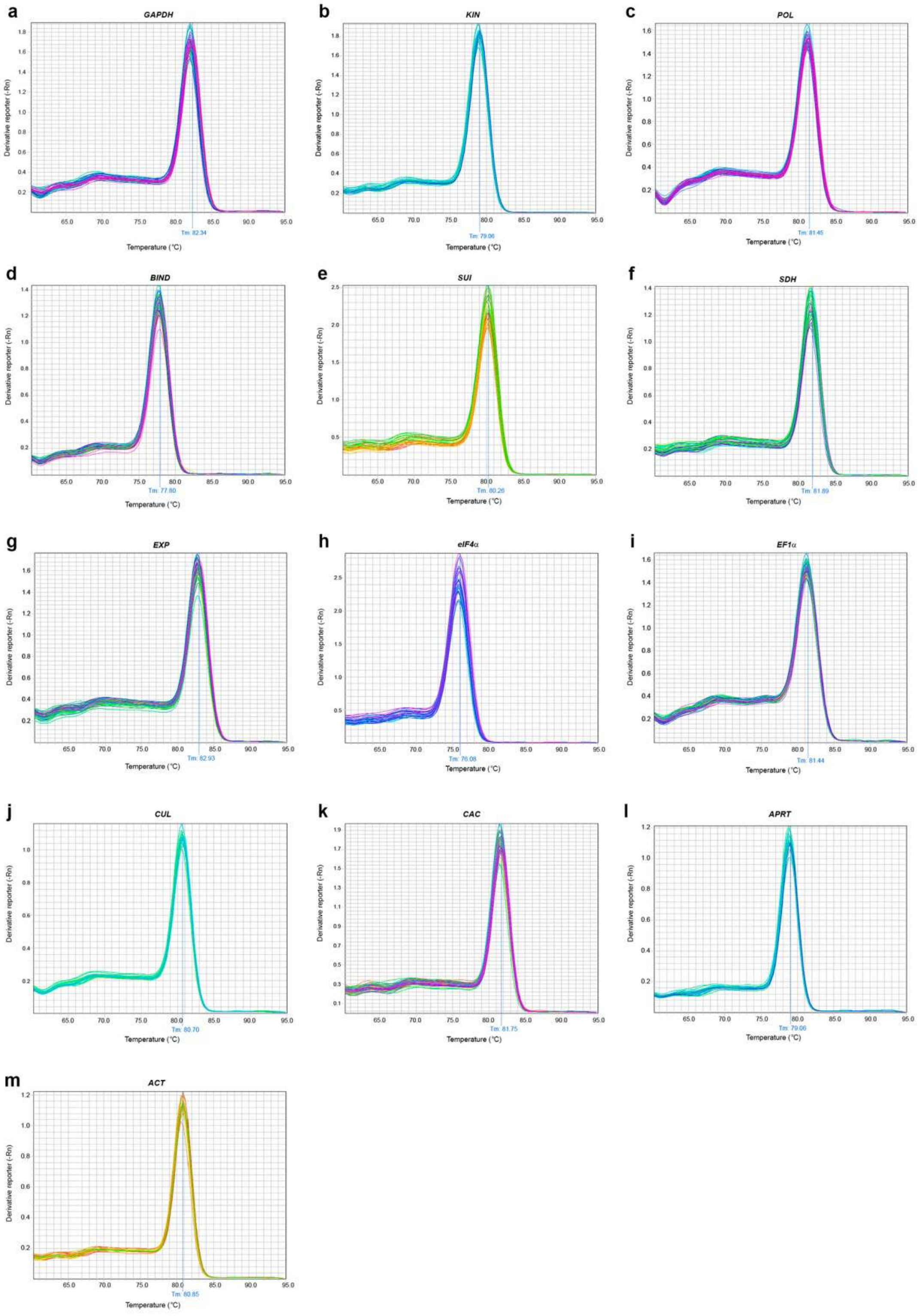

Figure S2: Dissociation curve data for the 13 reference genes tested. (**a**) *GAPDH*; (**b**) *KIN;* (**c**) *POL*; (**d**) *BIND*; (**e**) *SUI*; (**f**) *SDH*; (**g**) *EXP*; (**h**) *elF4α*; (**i**) *EF1α*; (**j**) *CUL*; (**k**) *CAC*; (**l**) *APRT* and (**m**) *ACT*.

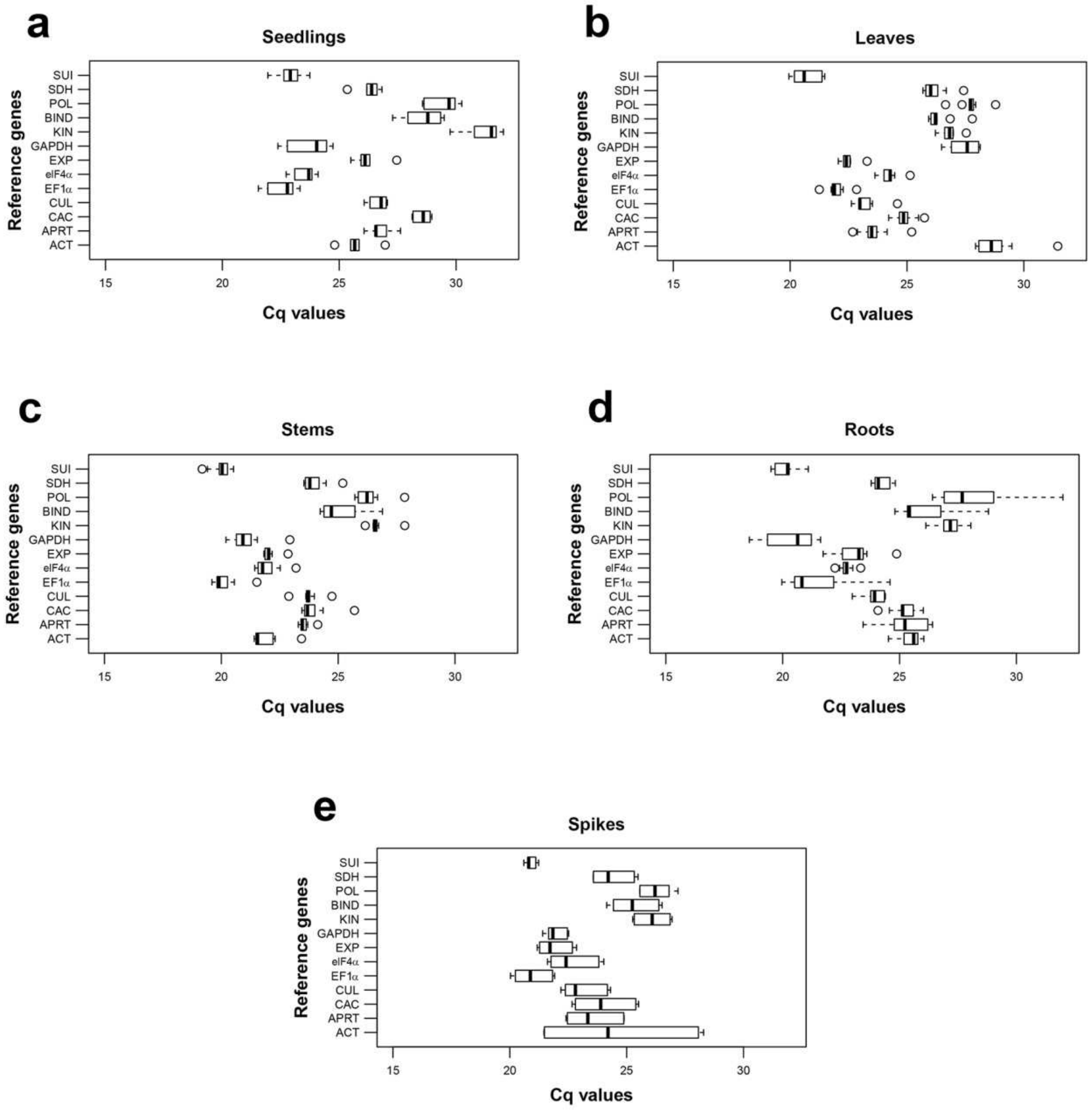

Figure S3: Expression level of reference genes tested in different tissues/organs. (**a**) Seedlings; (**b**) Leaves; (**c**) Stems; (**d**) Roots and (**e**) Spikes.

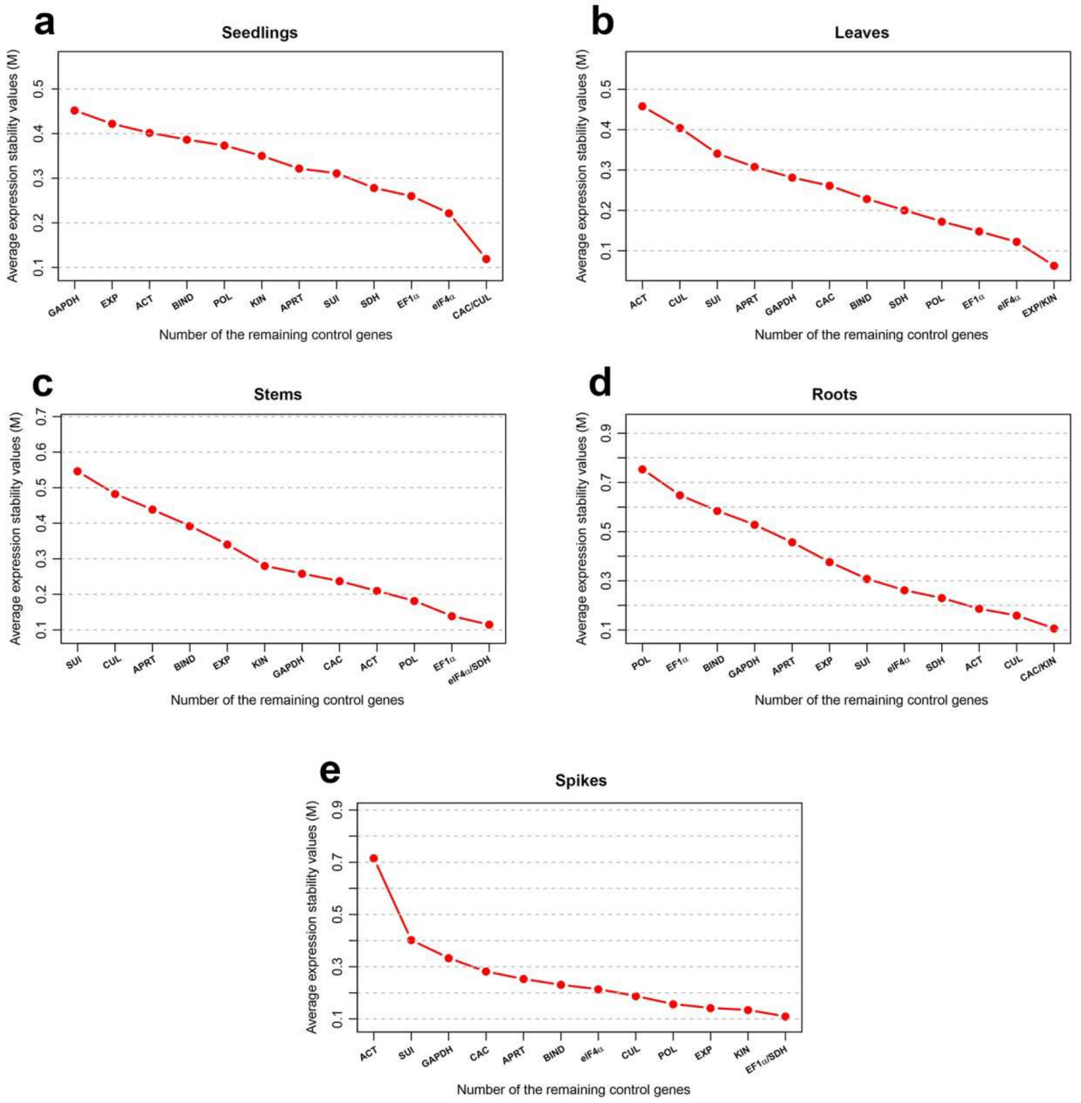

Figure S4: Average expression stability values (M) calculated by geNorm. A lower value of average expression stability (M) indicates most stable expression. (**a**) Seedlings; (**b**) Leaves; (**c**) Stems; (**d**) Roots and (**e**) Spikes.

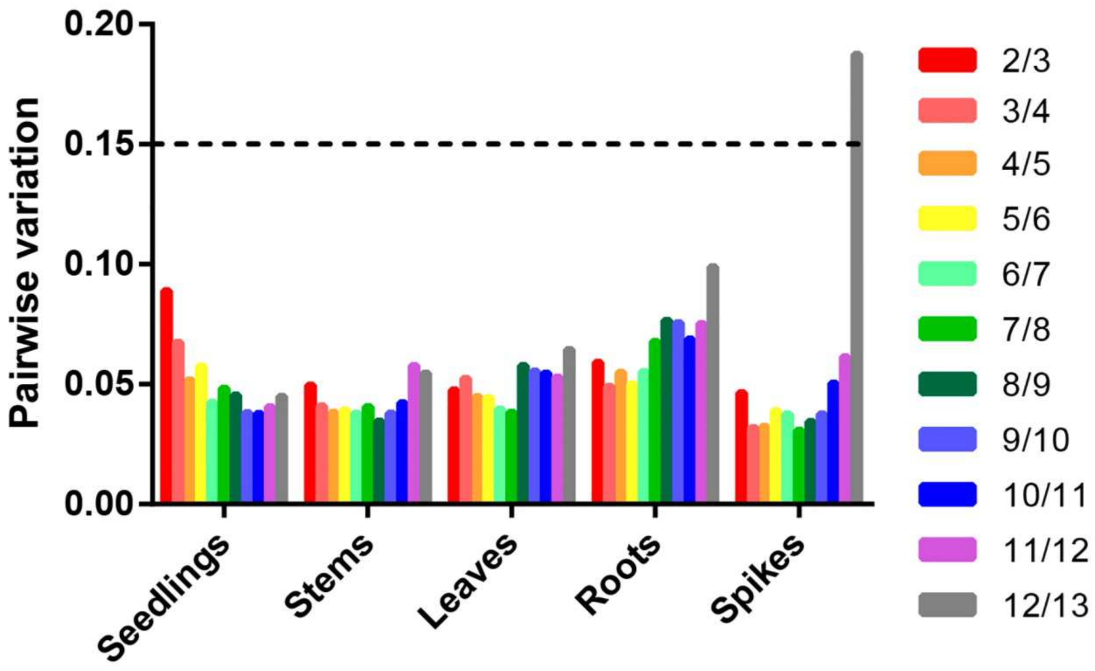

Figure S5: Pairwise variation (V) to define the optimal number of reference genes required to a more reliable normalization to seedlings, stems, leaves, roots and spikes.

Table S1: Information on reference and target genes used for qRT-PCR analysis

Gene symbol	Description	Source sequence ID	*S. viridis* Phytozome ID	E-value	*S. viridis* identity %	Primer sequences (5'-3')	Tm (°C)	Amplicon size (bp)	PCR Efficiency (%)	Reference
ACT	*Actin*	Si026509m	Sevir.8G042300	0	100	F: GCACCACCTGAGAGGAAATATAG<br>R: CTCATCGTACTCCGCCTTTG	72.1<br>70.2	102	92	[29]
eIF4α	Eukaryotic initiation factor 4-alpha	CA275432.1	Sevir.1G088000	0	100	F: GTCTGCTAAGGTGCTGGATAAA<br>R: ACCACTCCTCCAGAACATAGA	69.0<br>69.1	78	93	[27]
CUL	Cullin	CF574093.1	Sevir.3G038900	1.6e-121	93.1	F: TCTCATCACGAGGGACTACTT<br>R: CTTGCCAACAACCACCAATC	69.2<br>68.0	85	96	[27]
APRT	Anthranilate phosphoribosyl transferase	CA089592.1	Sevir.2G326600	0	100	F: GGCAACGGCTATGTTCCTAATA<br>R: CCTCATCCAGAAGAACCCAAG	69.1<br>71.0	103	93	[28]
CAC	Clathrin adaptor complex	CA203604.1	Sevir.1G284400	2.3e-54	100	F: CTGCTTCTGGTCTTCGTGTT<br>R: GTATGATCCTGCTCTCGTGATG	68.2<br>71.1	98	92	[27]
GAPDH	Glyceraldehyde 3-phosphate dehydrogenase	LOC_Os02g38920	Sevir.1G225700	2.1e-64	100	F: TGACAACAGGTCCAGCATCTTT<br>R: CGTACCAGGACACAAGCTTCAC	65.8<br>65.8	80	91	[31]
SUI	Translation factor SUI1	LOC_Os07g34589	Sevir.2G348300	2e-90	100	F: CCAGAGCTTGGACAGGTCATTC<br>R: ACAATGCCAGCCTGGACAA	67.1<br>66.8	80	90	[31]
KIN	Protein kinase	LOC_Os06g48970	Sevir.4G252700	5.8e-78	99.2	F: AAGTCCACTCGACCTTCAATCC<br>R: CTCAAGCGAACCGAATGAAAC	65.4<br>65.7	80	94	[31]
BIND	RNA-binding protein	LOC_Os03g46770	Sevir.9G090900	3.5e-89	71.9	F: CTCGCGGTTTCGGCTTT<br>R: CCAAGTCCAGTCCATTCATTCC	66.4<br>66.6	81	91	[31]
EF1α	Elongation Factor 1-alpha	Si022039m	Sevir.3G272400	0	96.1	F: TGGTATGCTTGTCACCTTTGGT<br>R: CTCGTGGTGCATCTCAACTGA	65.4<br>66.5	70	92	[29]
POL	RNA polymerase II	Si033113m	Sevir.2G148400	0	92.9	F: TTGAGGCTTCGTGCCATTG<br>R: CGGTAGCAGCTGGTGACCAT	67.3<br>67.5	70	93	[29]
SDH	Succinate dehydrogenase	Bradi1g26050	Sevir.7G038300	8.9e-83	100	F: ACAGTTCAGCGCTACGTTCT<br>R: GGTGGCTAGAGGGACGGATA	60.4<br>64.5	85	90	[30]
EXP	Expressed protein	LOC_Os07g02340	Sevir.9G362700	3.6e-15	47.8	F: GGAACGCCGAGAAGAACAAG<br>R: ACATACTTTGCACTGGATGCTCAT	66.0<br>65.6	83	92	[31]
GOLS2	Glycosyl transferase	AT1G56600	Sevir.2G450900	2.6e-107	99.7	F: CGAGGACATCAAGATGCTGGTGAG<br>R: TCCAGGCTCTCGTCGTTGAAGATG	71.0<br>72.4	62	95	[35]
P5CS	Δ^1-pyrroline-5-carboxylate synthase	AT2G39800	Sevir.5G386300	6.4e-43	100	F: TTGGTCTAGGTGCAGAGGTTGG<br>R: AGCCCATCAACACCAACAGGTC	67.4<br>69.3	73	100	[36]
SuSy	Sucrose Synthase	AF263384	Sevir.4G039300	9.9e-149	99.9	F: CTGACTGGTGTGTACGGATTC<br>R: CTTGCCAGGCTACGGTATTT	71.0<br>68.0	107	91	[57]

Table S2. *Setaria viridis* reference genes ranked according to expression stability as determined by geNorm.

Ranking	Seedlings		Leaf		Stem		Root		Spike	
	**geNorm**	**Stability value**	**geNorm**	**Stability value**	**geNorm**	**Stability value**	**geNorm**	**Stability value**	**geNorm**	**Stability value**
**1**	*CAC*	0.12	*EXP*	0.11	*eIF4α*	0.06	*CAC*	0.11	*CAC*	0.11
**2**	*CUL*	0.12	*KIN*	0.11	*SDH*	0.06	*KIN*	0.11	*KIN*	0.11
**3**	*eIF4α*	0.22	*eIF4α*	0.14	*EF1α*	0.12	*CUL*	0.16	*CUL*	0.16
**4**	*EF1α*	0.26	*EF1α*	0.18	*POL*	0.15	*ACT*	0.19	*ACT*	0.19
**5**	*SDH*	0.28	*POL*	0.21	*ACT*	0.17	*SDH*	0.23	*SDH*	0.23
**6**	*SUI*	0.31	*SDH*	0.24	*CAC*	0.20	*eIF4α*	0.26	*eIF4α*	0.26
**7**	*APRT*	0.32	*BIND*	0.26	*GAPDH*	0.23	*SUI*	0.31	*SUI*	0.31
**8**	*KIN*	0.35	*CAC*	0.28	*KIN*	0.26	*EXP*	0.38	*EXP*	0.38
**9**	*POL*	0.37	*GAPDH*	0.34	*EXP*	0.28	*APRT*	0.46	*APRT*	0.46
**10**	*BIND*	0.39	*APRT*	0.39	*BIND*	0.31	*GAPDH*	0.53	*GAPDH*	0.53
**11**	*ACT*	0.40	*SUI*	0.44	*APRT*	0.34	*BIND*	0.58	*BIND*	0.58
**12**	*EXP*	0.42	*CUL*	0.48	*CUL*	0.40	*EF1α*	0.65	*EF1α*	0.65
**13**	*GAPDH*	0.45	*ACT*	0.55	*SUI*	0.46	*POL*	0.75	*POL*	0.75
**Best pair**	***CAC/ CUL***		***EXP/ KIN***		***eIF4α/ SDH***		***CAC/ KIN***		***CAC/ KIN***	

Table S3. *Setaria viridis* reference genes ranked according to expression stability as determined by NormFinder.

Ranking	Seedlings		Leaf		Stem		Root		Spike	
	**Norm Finder**	**Stability value**	**Norm Finder**	**Stability value**	**Norm Finder**	**Stability value**	**Norm Finder**	**Stability value**	**Norm Finder**	**Stability value**
**1**	*SDH*	0.01	*EXP*	-0.003	*SDH*	0.004	*EXP*	0.03	*eIF4a*	-0.02
**2**	*EF1α*	0.01	*KIN*	0.002	*eIF4α*	0.007	*ACT*	0.05	*BIND*	-0.01
**3**	*eIF4α*	0.02	*eIF4α*	0.004	*EF1α*	0.009	*CUL*	0.06	*SDH*	-0.01
**4**	*CAC*	0.03	*SDH*	0.011	*EXP*	0.015	*CAC*	0.06	*CUL*	-0.01
**5**	*CUL*	0.04	*BIND*	0.021	*KIN*	0.021	*KIN*	0.07	*APRT*	-0.01
**6**	*SUI*	0.04	*EF1α*	0.024	*POL*	0.025	*SDH*	0.07	*EF1α*	-0.01
**7**	*KIN*	0.04	*POL*	0.028	*ACT*	0.026	*eIF4α*	0.10	*KIN*	0.02
**8**	*APRT*	0.05	*CAC*	0.040	*CAC*	0.043	*SUI*	0.11	*CAC*	0.02
**9**	*BIND*	0.07	*GAPDH*	0.137	*GAPDH*	0.059	*BIND*	0.15	*EXP*	0.02
**10**	*POL*	0.07	*APRT*	0.167	*APRT*	0.061	*APRT*	0.17	*POL*	0.04
**11**	*ACT*	0.08	*SUI*	0.180	*BIND*	0.118	*EF1α*	0.29	*GAPDH*	0.20
**12**	*EXP*	0.13	*CUL*	0.182	*CUL*	0.200	*GAPDH*	0.30	*SUI*	0.40
**13**	*GAPDH*	0.16	*ACT*	0.331	*SUI*	0.239	*POL*	0.78	*ACT*	2.85
**Best pair**	***SDH/ EF1α***		***SDH/ KIN***		***eIF4α/ EXP***		***CUL/ KIN***		***eIF4α/ APRT***	
